# Differences in Milk Fatty Acids Profile of Two Breeds of Water Buffaloes Explained by Their Gastrointestinal Microbiota

**DOI:** 10.3390/ani14152146

**Published:** 2024-07-23

**Authors:** Yameng Zhao, Yanxia Guo, Chengjian Yang, Ziyi Song, Xianqing Luo

**Affiliations:** 1 Guangxi Key Laboratory of Animal Breeding, Disease Control and Prevention, College of Animal Science and Technology, Guangxi University, Nanning 530004, China; aiyoyo198@126.com; 2 Guangxi Key Laboratory of Buffalo Genetics, Reproduction and Breeding, Guangxi Buffalo Research Institute, Nanning 530023, China; gyxlq0417@163.com (Y.G.); 19101477179@163.com (X.L.)

**Keywords:** water buffalo, milk fatty acid, breed, rumen microbiota, fecal bacteria

## Abstract

**Simple Summary:**

Buffalo milk has a significantly greater fatty acid content, up to 11%, than cow’s milk, making buffalo milk more suitable for dairy products such as cheese. Several factors affect the quantity and proportion of milk fatty acids, and among the intrinsic factors, breed is important. However, the role of breed is still not fully understood. It is known that gastrointestinal microbiota serve as a key factor in modulating the content of milk fatty acids, since breed affects the type of gastrointestinal microbiota and consequently the milk fatty acids (FA). Therefore, we used 16S rDNA sequencing to analyze the microbial causes of different milk fatty acids in two breeds of dairy buffaloes. The results showed that the abundance of fatty acids such as C18:3 and C18:2c9t11 in Murrah buffalo milk was associated with the abundance of bacteria such as *Acetobacter* and *Ruminalococcus* in the rumen, as well as bacteria such as UCG-005 in the feces.

**Abstract:**

This experiment investigated gastrointestinal microbes’ role in milk fatty acid differences between Murrah and Nili-Ravi buffaloes. After 30 days of a basal diet, rumen microbial diversity was similar, but Murrah buffaloes had greater partially unsaturated fatty acids like C18:2c9t11. Rumen bacteria like *Acetobacter*, *Ruminococcus*, and *Prevotellaceae*_YAB2003_group correlated positively with milk fatty acids C22:5n-6 and C18:3 in Murrah. Fecal microbial beta diversity differed, with UCG-005 and *Prevolla* positively correlated with C18:2c9t11 and C22:5n-6. The greater quantity of milk fatty acids C18:3, C18:2c9t11, and C22:5n-6 in Murrah milk was linked to rumen and fecal microbes. This suggests that gastrointestinal microbes like *Acetobacter*, *Ruminococcus*, and UCG_005 regulate milk fatty acid concentrations in buffaloes.

## 1. Introduction

As the second largest source of milk in the world [1,2], water buffalo accounts for about 50% of the total milk production in Asia [3]. Compared with cow milk, water buffalo milk is more abundant in fat, protein, amino acids, and unsaturated fatty acids, and is lower in cholesterol concentrations [4,5,6]. Therefore, water buffalo milk and its dairy products are becoming increasingly popular among consumers. In some Asian countries such as India, fresh buffalo milk accounts for more than half of liquid milk consumption, and buffalo dairy products such as butter, ghee, and yogurt are also widely consumed [7]. In some Mediterranean countries in Europe, the buffalo industry is more developed. Buffalo milk is mainly used to produce various dairy products such as butter, cheese, and ice cream, while Italian buffalo milk is almost exclusively used to produce mozzarella cheese [8,9].

The fatty acid content of buffalo milk is very rich, which is of great significance for human nutrition and health. Over the past 30 years, many people have continued to focus on and study the role of fatty acids, and it has been reported that dietary supplementation with short-chain fatty acids can prevent and treat insulin resistance [10], have immunotherapeutic effects in cellular carcinogenesis [11], facilitate insulin sensitivity [12], and contribute to dietary, intestinal flora and energy metabolism [13]. Linoleic acid (LA) and α-linolenic acid (ALA) in long-chain fatty acids (LCFA) are essential fatty acids for the human body and precursors for the synthesis of conjugated linoleic acid (CLA). The human body cannot synthesize CLA, but it can be obtained from milk and meat. Den Hartigh et al. [14,15,16,17,18] have demonstrated that conjugated linoleic acid present in milk possesses numerous physiological benefits, including anti-mutagenic properties, antioxidant activity, anti-tumor effects, antibacterial capabilities, cholesterol-lowering in humans, anti-atherosclerotic benefits, management of diabetes mellitus, and enhancement of growth. It has furthermore been documented that the supplementation of α-linolenic acid at a specific concentration exhibits a markedly greater impact on the amelioration of cardiovascular disease, and it impedes the proliferation and spread of osteosarcoma cells by suppressing the expression of fatty acid synthase (FASN). This finding lays the groundwork for potential therapeutic targets in the management of osteosarcoma [19,20]. It has been shown that 20% of the fatty acids in milk come from the rumen [21], and microbial abundance is higher in cows with greater saturated fatty acid (SFA) content in milk [22]. Ruminal microorganisms are the source of many bioactive fatty acids in ruminant-derived foods, and rumen bacteria biohydrogenate a variety of intermediates that play an important role [23], and feed is broken down by rumen bacteria into VFA. This process also produces long-chain fatty acids that are absorbed by the animal [24]. Odd-chain and branched-chain fatty acids in milk are biomarkers of rumen fermentation and are closely related to the fermentation pattern of the rumen [25,26].

As an important component of buffalo milk, the metabolism of fatty acids varies with a series of related factors such as breed, season, and feed. We speculate that there are differences in fatty acids between two breeds of water buffalo, which may also be related to differences in rumen microbiota. The current research has focused on regulating the nutrient content of milk through feed additives [27,28], while relatively few studies have explored the differences in fatty acid content in the milk of different breeds of buffalo from the perspective of gastrointestinal microorganisms. Due to the poor milk production of Chinese indigenous buffaloes [29], we chose Nili-Ravi and Murrah dairy buffaloes as the study subjects to explore the effect of gastrointestinal microorganisms on the difference in milk fatty acids in the two types of buffalo milk, which provides a new idea for regulating the content of milk fatty acids and other nutrients in buffalo milk by microbial means.

## 2. Materials and Methods

### 2.1. Diet and Animal Management

Twenty-four healthy, lactating Murrah buffaloes and Nili-Ravi buffaloes (12 of each breed; initial body weight, 615 ± 21 kg; lactation stage, 135 ± 21.5 d; milk yield 7.85 ± 1.48 kg; parity, 2.5 ± 0.5) were selected for this study. The used study design was a completely randomized design with two treatments (breeds of dairy buffaloes) and 12 dairy buffaloes for each breed. The experiment spanned a duration of 30 days, preceded by a 7-day pre-feeding phase. Daily management of buffaloes was carried out according to the established procedures of the buffalo farm in Guangxi Buffalo Research Institute. During feeding time, all animals were housed in individual stalls with water sinks, a concrete floor, shade shelter, and external exercise yards. The diets were offered twice daily at 06:00 h and 16:00 h. All water buffaloes had free access to freshwater during the entire experimental period. Water buffaloes were milked twice daily (at 06:00 and 16:00). Milking was performed with a milking machine. The diet’s ingredients and nutrient composition are shown in Table 1. The composition was determined as follows: dry matter was determined according to the methods of AOAC [30]; crude protein content was determined by Kjeldahl nitrogen determination; and neutral detergent fiber (NDF) and acid washing fiber were determined according to the Van Soest method [31].

### 2.2. Measurement of Dry Matter Consumption, Milk Production, Milk Constituents, and Milk Fatty Acids Profile

Feed intake was measured during the last three days of experimental period. Water buffaloes were milked twice daily (at 06:00 h and 16:00 h) and the milk yield was recorded every day. During the last three days of the experiment period, 100 mL milk samples were collected from each buffalo. A quantity of 50 mL of milk was sampled for analysis of its composition, encompassing protein, fat, lactose, and total solids content. This analysis was conducted using a milk composition analyzer, specifically the FOSS MilkScan FT120 from Foss Electric, a Danish company under the Hildesforth Electric umbrella. The leftover milk samples were thoroughly combined in equal volumes, from which 50 mL was extracted into a centrifuge tube and dispatched to Shanghai Minxin Biotechnology Co. (Shanghai, China) for evaluation of the milk’s fatty acid composition. For specific test methods refer to Bai et al. [32]. Fatty acids were determined on a Thermo Trace 1300 gas chromatograph-ISQ7000 mass spectrometer (GC-MS); chromatographic column: DB-5ms (60 m × 0.25 mm × 0.25µm). Methods of pre-treatment of milk samples: Place 0.2 mL of sample in a 20 mL glass centrifuge tube, add 5 mL of 10% acetyl chloride methanol and 1 mL of n-hexane, and 5 μL of internal standard (C19:0,10 mg/mL). React at 95 °C for 4 h at 250 rpm. Add 6 mL of 6% potassium carbonate solution and vortex for 2 min. Centrifuge to remove the n-hexane layer and perform rotary evaporation to remove the n-hexane. Add 400 μL of n-hexane and vortex for 1 min. The supernatant was centrifuged at 12,000 rpm for 5 min and placed in a vial for examination.

### 2.3. Assessment of Rumen Fermentation Parameters, along with Fatty Acids Profiling

Buffaloes were fasted for 12 h before ruminal sampling. The rumen contents (500 mL, comprising both solid and liquid phases) were solely gathered on the final day of the experiment, prior to the morning feeding session, employing a stomach tube [33]. Following collection, the samples were promptly delivered to the laboratory. Approximately 8 mL of the rumen fluid was utilized to quantify the microbial protein (MCP) content via colorimetry, utilizing a UV-Vis spectrophotometer (model PE lambda 35, Shanghai Pudi Biotechnology Co., Ltd., Shanghai, China) [34]. To determine the ammonia nitrogen (NH_3_-N) level, 4 mL of rumen fluid was combined with 4 mL of 0.2 mol HCl and stored at −20 °C for future analysis. Subsequently, the NH_3_-N concentration was assessed using the indophenols method on a UV-Vis spectrophotometer (PE lambda 35, Shanghai Pudi Biotechnology Co., Ltd., China) at a wavelength of 560 nm [35]. The volatile fatty acid (VFA) content was analyzed by mixing 1 mL of rumen fluid with 0.5 mL of 8.2% metaphosphoric acid, followed by centrifugation at 20,000× *g* for 10 min at 4 °C. Then, 920 µL of the supernatant was combined with 80 µL of an internal standard, crotonic acid (1 mol/L). The various VFA fractions were quantified using a gas chromatography (GC) system, as described previously [36]. The fatty acid content in rumen contents (including rumen fluid and feed pellets) was sent to Shanghai Minxin Biotechnology Co., Ltd. for testing. The pre-treatment for the determination of fatty acids in rumen fluid is similar to the pre-treatment for milk fatty acids.

### 2.4. Feces Collection

On the last day of the experiment, feces samples of the experimental buffalo were collected by rectal palpation (*n* = 24). The buffaloes were subjected to rectal palpation to collect feces, which was then transferred to a sterile 50 mL centrifuge tube and immediately placed on ice [37]. Samples were stored at −20 °C for further analysis.

### 2.5. 16S rDNA Gene Sequencing and Bioinformatic Analysis

Rumen fluid and fecal specimens were retrieved from the −20 °C freezer and allowed to defrost at 4 °C, and 1 mL of the thawed rumen/fecal material (both solid and liquid components) was subjected to DNA extraction employing the CTAB bead-beating technique [38]. The purity and quantity of DNA were assessed using a Nanodrop spectrophotometer (Nanodrop ND-2000, Beijing Xinxing Johnson Biotechnology Co., Beijing, China). The extracted DNA samples were sent to Shanghai Ouyi Co. (Shanghai, China) for sequencing. Primers “343F: TACGGRAGGCAGCAG; 798R: AGGGTATCTAATCCT” were used to amplify the V3 and V4 regions of 16S RNA, respectively [39]. High-throughput sequencing was performed and raw data were obtained using the Illumina MiSeq sequencing platform, and the raw reads data volume was distributed around 80,000. We used fastp to cut out primer sequences from raw data sequences. The representative sequences and ASV abundance tables were derived through rigorous quality assurance measures, including quality filtering, noise suppression, sequence assembly, chimera removal, and other default parameter-guided quality control analyses of the qualified paired-end raw data from the preceding step. This was accomplished utilizing Qiime2 (version 2020.11) within the DADA2 analytical framework. ASV abundance was obtained by comparing each species with the silva (version 138) database and classifying and categorizing each species. Utilizing a cloud-based platform, the ASV data underwent binary analysis to assess relative abundance, microbial diversity indices, and various other pertinent metrics.

### 2.6. Statistical Analysis

The experimental results are presented as mean values ± standard deviations. The data were statistically analyzed employing one-way analysis of variance (ANOVA) in the SPSS software package (SPSS, 16.0). A *p*-value less than 0.05 was considered statistically significant. Microbial distributions at the alpha diversity index, beta diversity, phylum level, and genus level were analyzed using the vegan and phyloseq packages in R (4.2.3). The primary bacterial groups serving as biomarkers in each treatment cohort were identified through linear discriminant analysis (LDA) effect size (LEfSe). For this study, bacterial taxa with LDA scores (on a log10 scale) exceeding 4 were deemed significantly distinct. To assess the relationship between rumen fermentation and fatty acid characteristics with the top 30 genera of the microbiota, Spearman correlation analysis (r) was employed. The presence of an asterisk indicated a *p* value less than 0.05 (* *p* < 0.05, ** *p* < 0.01, *** *p* < 0.001).

## 3. Results

### 3.1. Milk Yield, Milk Composition, and Milk Fatty Acids

The breed did not significantly influence the average dry matter intake, milk production, or milk composition (*p* > 0.05; refer to Table 2).

The concentrations of fatty acids are shown in Table 3. The concentrations of milk fatty acids C18:3, C18:2c9t11, and C22:5n-6 were greater in Murrah buffaloes (*p* < 0.05). Conversely, higher concentrations of C11:0, C18:1, and C18:0 fatty acids in milk were found in Nili-Ravi buffaloes (*p* < 0.05). No significant difference was found in the concentrations of other fatty acids (*p* > 0.05).

### 3.2. Rumen Fermentation Characteristics, and Ruminal Fatty Acids

The rumen fluid fermentation metrics are presented in Table 4, indicating decreased pH levels and a diminished acetic acid/propionic acid ratio in the Murrah buffaloes. Conversely, elevated levels of NH_3_-N, acetic acid, propionic acid, isovaleric acid, valeric acid, and total volatile fatty acids were observed (*p* < 0.05). However, no notable variations were noted in other rumen fermentation indicators (*p* > 0.05).

The fatty acid concentrations in the rumen fluid are provided in Table 5. The concentrations of C15:0, C17:0, C18:2, C18:2c9t11, C18:2c12t10, C18:1t, C20:4n6, C20:2, C21:0, C24:0, UFA, MUFA, Omega-3, Omega-6, Omega-9, and PUFA in the rumen contents of Murrah buffaloes were higher. However, the concentrations of C12:0, C17:1, C18:3, C18:0, C22:3, C20:3, and C20:0, and the SFA/UFA ratio, were lower in Nili-Ravi buffaloes (*p* < 0.05). The concentrations of other fatty acids did not exhibit significant variations (*p* > 0.05).

### 3.3. Ruminal Bacterial Communities

#### 3.3.1. Alpha and Beta Diversity Assessments, along with the Proportional Abundance of Ruminal Bacterial Communities

**Figure 1:**
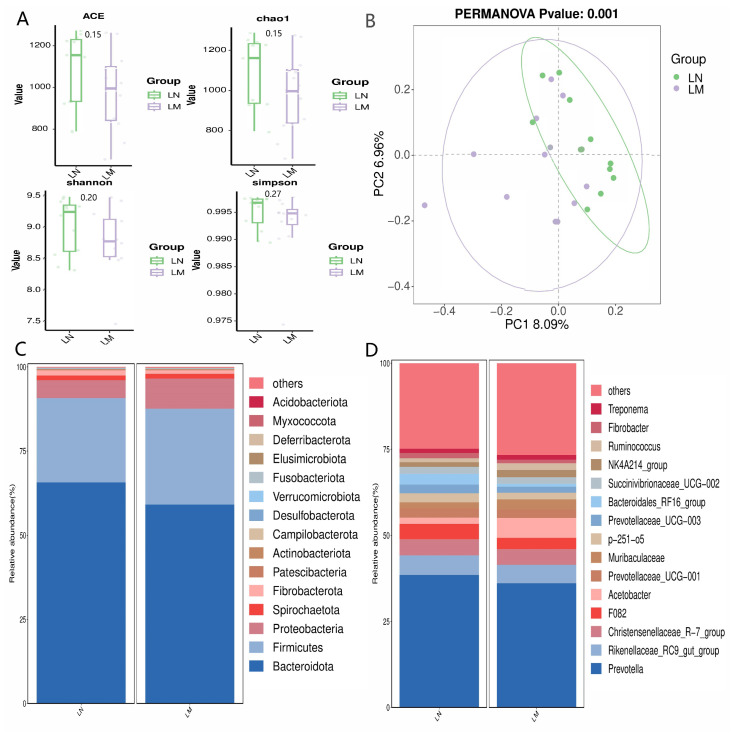
Ruminal bacterial communities in two breeds of water buffalo. (**A**) Ruminal bacterial alpha diversity parameters. (**B**) Principal coordinate analysis (PCoA) of taxonomic classifications across distinct treatment groups. (**C**) Phylum-level abundance of buffalo rumen microbiota. (**D**) Genus-level abundance of buffalo rumen microbiota. LM: Murrah buffalo rumen fluid group; LN: Nili-Ravi buffalo rumen fluid group.

The graphical representation of alpha and beta diversity analysis, along with the relative abundance of ruminal bacterial populations, is presented in Figure 1. The sequencing analysis achieved a coverage index surpassing 99%, indicating that the sequencing outcomes accurately mirror the actual state of the ruminal microbiome. No significant disparities were observed in terms of microbial richness (ACE and Chao1 indices) and diversity (Shannon and Simpson indices) between the two groups of rumen fluid samples (*p* > 0.05, Figure 1A). The microbial composition in the two groups of rumen fluid was relatively similar (Figure 1B). The *Bacteroidetes*, *Firmicutes*, and *Proteobacteria* bacterial groups constituted the primary microbial populations in the buffalo rumen, comprising over 86% of the entire microbial community (Figure 1C). At the genus level, the buffalo rumen microflora consisted mainly of *Prevotella*, *Rikenellaceae*_RC9_gut_group, *Christensenellaceae*_R-7_group, and others (Figure 1D).

**Figure 2:**
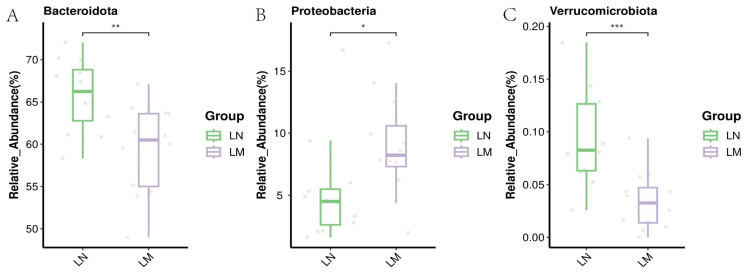
Relative abundance of differentially abundant bacterial phyla (* 0.01 < *p* ≤ 0.05, ** 0.001 < *p* ≤ 0.01, *** *p* ≤ 0.001). (**A**) *Bacteroidota*; (**B**) *Proteobacteria*; (**C**) *Verrucomicrobiota*. LM: Murrah buffaloes rumen fluid group; LN: Nili-Ravi buffaloes rumen fluid group.

The figure depicted in Figure 2 illustrates the comparative abundance of rumen-specific bacterial phyla. Additionally, other prevalent bacterial phyla include *Actinobacteriota*, *Cyanobacteria, Spirochaetota*, and *Verrucomicrobia*. Within the rumen of Nili-Ravi buffaloes, *Bacteroidota* and *Verrucomicrobiota* exhibited greater relative abundances (*p* < 0.01, Figure 2A,C), whereas the Murrah buffaloes’ rumen showed a higher relative abundance of *Proteobacteria* (*p* < 0.05, Figure 2B).

**Figure 3:**
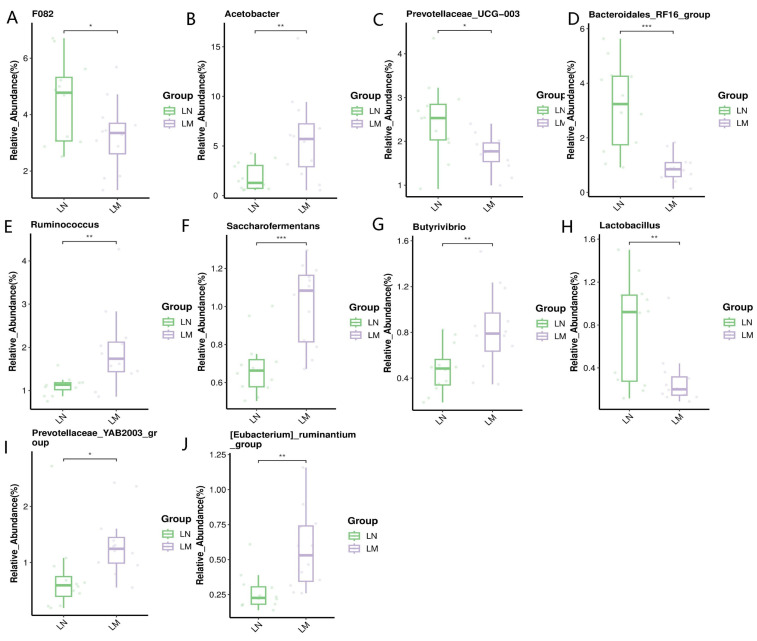
Relative abundance at the genus level of differentially abundant rumen bacteria, (* 0.01 < *p* ≤ 0.05, ** 0.001 < *p* ≤ 0.01, *** *p* ≤ 0.001). (**A**) F082; (**B**) *Acetobacter;* (**C**) *Prevotellaceae*_UCG_003; (**D**) *Bacteroidales*_RF16_group; (**E**) *Ruminococcus*; (**F**) *Saccharofermentans*; (**G**) *Butyrivibrio*; (**H**) *Lactobacillus*; (**I**) *Prevotellaceae*_YAB2003_group; (**J**) [*Eubacterium*]_*ruminantium*_group. LM: Murrah buffalo rumen fluid group; LN: Nili-Ravi buffalo rumen fluid group.

The relative abundance of differentially abundant bacterial genera is shown in Figure 3. At the genus level, the relative abundances of *Acetobacter*, *Ruminococcus*, *Saccharoferments*, *Butyrivibrio*, *Prevotellaceae*_YAB2003_Group, and [*Eubacterium*]_*Ruminantium*_group in the rumen of Murrah buffaloes were higher (*p* < 0.05, Figure 3B,E–G,I,J); and the relative abundances of F082, *Prevotellaceae*_UCG_003, *Bacteroidales*_RF16_group, and *Lactobacillus* of the rumen of Nili-Ravi buffaloes were higher (statistically significant at *p* < 0.05, Figure 3A,C,D,H).

#### 3.3.2. Biomarker Bacteria Taxa

**Figure 4:**
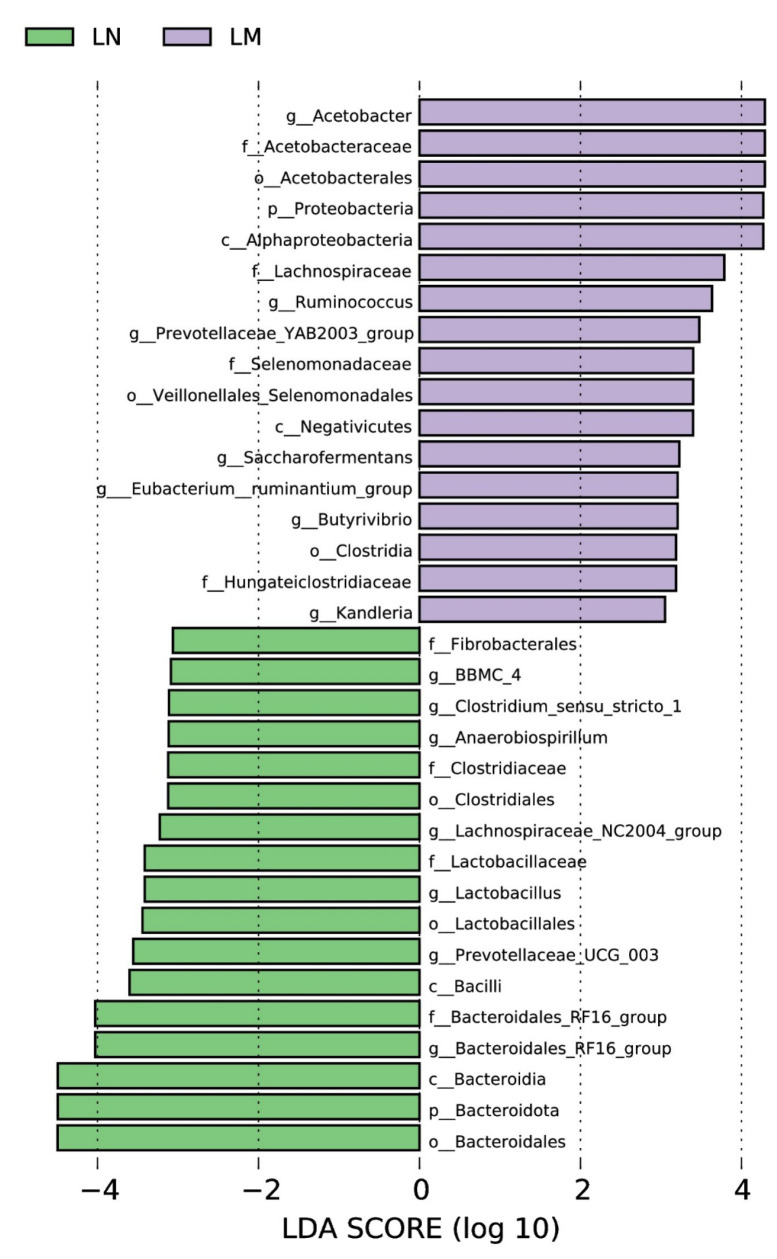
Distinctive bacterial genera in various treatment groups, as identified through linear discriminant analysis (LDA) effect size (LEfSe) analysis, exhibiting an LDA score greater than 4.0, serve as biomarkers.

Biomarker bacteria taxa is shown in Figure 4. Utilizing LEfSe, we pinpointed bacterial taxa exhibiting marked abundance as distinguishing biomarkers among the treatment groups. A cumulative total of 10 significant taxonomic clades, each with an LDA score exceeding 4.0, were discerned, inclusive of two genera biomarkers. Notably, *Acetobacter* emerged as a biomarker in the rumen of Murrah buffaloes, whereas *Bacteroidales*_RF16_groups was identified as a biomarker in the rumen of Nili-Ravi buffaloes.

#### 3.3.3. Association of Rumen Bacteria with Ruminal Fermentation Parameters and Milk Fatty Acid Contents

**Figure 5:**
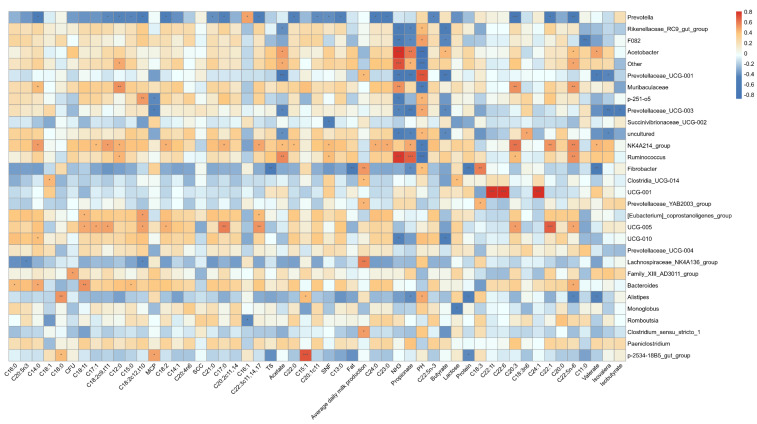
Association analyses were performed between ruminal fermentation parameters, milk fatty acid profiles, and the top 30 rumen bacterial genera. The two-dimensional heatmap visualizes the variation in color and its intensity, representing the type and magnitude of the correlation, respectively. The asterisk notation signifies statistical significance with *p* values less than 0.05 (* *p* < 0.05, ** *p* < 0.01, *** *p* < 0.001).

The Spearman’s correlation matrix between the relative abundance of rumen bacterial genera and fermentation parameters, as well as milk fatty acids, is depicted in Figure 5. *Prevotella* displayed a positive correlation with C16:1 but a negative correlation with C14:0, C12:0, C15:0, C21:0, C22:0, C13:0, C24:0, C23:0, C18:2c9t11, C18:2c12t10, C18:2, C17:0, C22:3c11 14 17, C20:1c11, SNF, C22:5n3, C20:3, and C22:5n6, all with statistical significance at *p* < 0.05. *Rikenellaceae*_RC9_gut_group and F082 were positively correlated with pH but negatively correlated with acetate, NH_3_-N, propionate, and butyrate (*p* < 0.05). *Acetobacter* was negatively correlated with pH but positively correlated with acetate, NH_3_-N, propionate, butyrate, C22:5n6, and valerate (*p* < 0.05). *Prevotellaceae*_UCG_001 was negatively correlated with valerate but positively correlated with pH and average daily milk production (*p* < 0.05). *Muribaculaceae* exhibited a negative correlation with pH but a positive correlation with C14:0, C12:0, NH_3_-N, C20:3, and C22:5n6, all of which were statistically significant at *p* < 0.05. P-251-05 was negatively correlated with C18:2c9t10 and NH_3_-N but positively correlated with MCP and pH (*p* < 0.05). *Prevotellaceae*_UCG_003 was negatively correlated with MCP, isovalerate, and isobutyrate (*p* < 0.05). *Succinivibrionaceae*_UCG_002 was negatively correlated with SNF (*p* < 0.05). NK4A214_group was negatively correlated with pH but positively correlated with C14:0, C17:1, C18:2c9t11, C12:0, C18:2, C17:0, C22:3c11 14 17, acetate, C22:0, SNF, C24:0, C23:0, C20:3, C22:1, C22:5n-6, and valerate (*p* < 0.05). *Ruminococcus* was negatively correlated with pH but positively correlated with C12:0, acetate, NH_3_-N, propionate, C20:3, and C22:5n6 (*p* < 0.05). *Fibrobacter* and *Prevotellaceae*_YAB2003_group were positively correlated with average daily milk production and C18:3 (*p* < 0.05). *Fibrobacter* was negatively correlated with milk fat, milk protein, TS, and valerate (*p* < 0.05). *Clostridia*_UCG-014 showed a positive correlation with C18:1, lactose, and average daily milk production *(p* < 0.05). UCG-001 was positively correlated with C22:1t, C22:6, and C24:1 (*p* < 0.05). [*Eubacterium*]_*coprostanoligenes*_group was positively correlated with C18:2c9t10, C18:1t, and C22:3c11 14 17 (*p* < 0.05). UCG-005 was positively correlated with C18:1t, C17:1, C18:2c9t11, C18:2c12t10, C18:2, C17:0, C22:3c11 14 17, C20:3, C22:1, and C22:5n6 (*p* < 0.05). UCG-010 was positively correlated with C15:0 but negatively correlated with NH_3_-N and butyrate (*p* < 0.05). *Lachnospiraceae*_NK4A136_group was negatively correlated with C20:5n3, and C18:2c12t10 (*p* < 0.05). *Bacterodes* was positively correlated with C16:0, C14:0, C18:1t, C15:0, and C22:5n6 (*p* < 0.05). *Alistipes* was positively correlated with C18:0, C15:1, and pH but negatively correlated with milk protein, C22:5n6, and valerate (*p* < 0.05). *Monoglobus* was negatively correlated with lactose (*p* < 0.05). *Romboutsia* was negatively correlated with C16:1 (*p* < 0.05). *Clostridium*_*sensu*_*stricto*_1 was positively correlated with average daily milk production (*p* < 0.05). P-2534-18B5_gut_group was positively correlated with C18:0, C15:1, and MCP but negatively correlated with milk protein (*p* < 0.05).

### 3.4. Fecal Bacterial Communities

#### 3.4.1. Alpha and Beta Diversity Analysis, and Relative Abundance of Fecal Bacterial Populations

**Figure 6:**
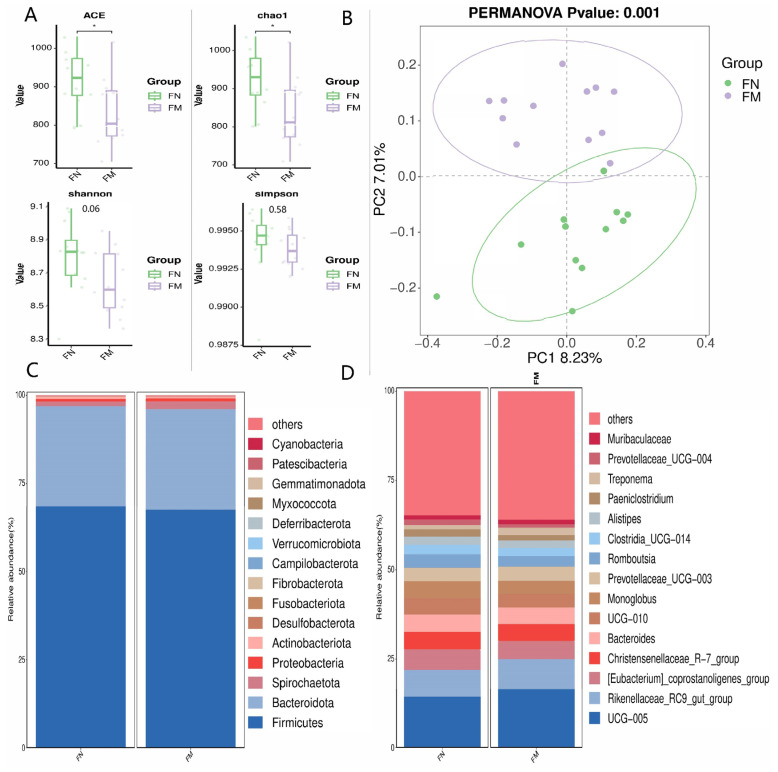
Fecal bacterial communities in two breeds of water buffalo. (**A**) Fecal bacterial alpha diversity parameters. (**B**) PCoA analysis of taxonomical classifications in different treatment groups. (**C**) Relative abundance of fecal microflora of buffalo at the phylum level. (**D**) Relative abundance of fecal microflora of buffalo at the genus level. * 0.01 < *p* ≤ 0.05.

Alpha and beta diversity analysis, as well as the relative abundance of fecal bacterial populations, are shown in Figure 6. The coverage index of this sequencing analysis was above 99% and close to 1, which means that the sequencing results can reflect the real situation of the fecal microbiome. Compared with Murrah buffaloes (FM), the ACE and Chao1 indexes in feces of Nili-Ravi buffaloes (FN) increased (*p* < 0.05). The Shannon and Simpson indices of the two groups exhibited no noteworthy divergence (*p* > 0.05, Figure 6A). The findings revealed certain variations in the composition of fecal microbiota between the Murrah buffaloes (FM) and Nili-Ravi buffaloes (FN) (*p* = 0.001, Figure 6B). At the phylum level, the main dominant microbial communities of the two groups of fecal microorganisms were *Firmicutes* and *Bacteroidetes* (Figure 6C). At the genus level, the main dominant bacteria of the two microbial groups are UCG_005, *Rikenellaceae*_RC9_gut_group, [*Eubacterium*]_*Coprostanoligenes*_group, *Christensenellaceae*_R-7_group, *Bacteroides*, UCG_010, etc. (Figure 6D).

**Figure 7:**
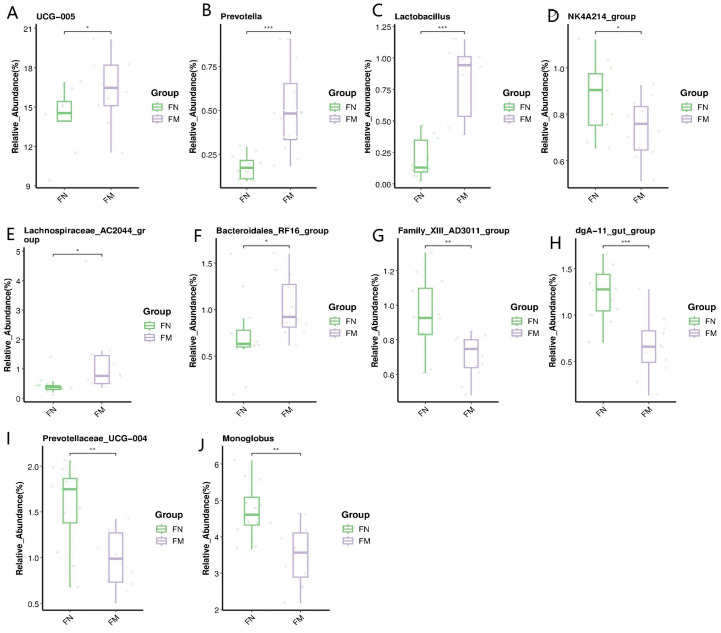
Relative abundance at the genus level of fecal differential bacteria, (* 0.01 < *p* ≤ 0.05, ** 0.001 < *p* ≤ 0.01, *** *p* ≤ 0.001). (**A**) UCG_005; (**B**) *Prevotella;* (**C**) *Lactobacillus*; (**D**) Nk4a214_group; (**E**) *Lachnospiraceae*_AC2044_group; (**F**) *Bacteroidales*_RF16_group; (**G**) Family_XIII_AD3011_group; (**H**) dgA_11_gut_group; (**I**) *Prevotellaceae*_UCG_004; (**J**) *Monoglobus*.

The percentage representation of fecal microbiota in water buffalo, stratified by phylum, is displayed in Figure 7. At the genus level, the relative abundances of UCG_005, *Prevotella*, *Lactobacillus*, *Lachnospiraceae*_AC2044_group, and *Bacteroidales*_RF16_group in FM group were higher (*p* < 0.05, Figure 7A–C,E,F). However, the relative abundance of Nk4a214_group, Family_XIII_AD3011_group, dgA_11_Gut_Group, *Prevotellaceae*_UCG_004, and *Monoglobus* in FM group were lower (*p* < 0.05, Figure 7D,G–J).

#### 3.4.2. Biomarker Bacteria Taxa

**Figure 8:**
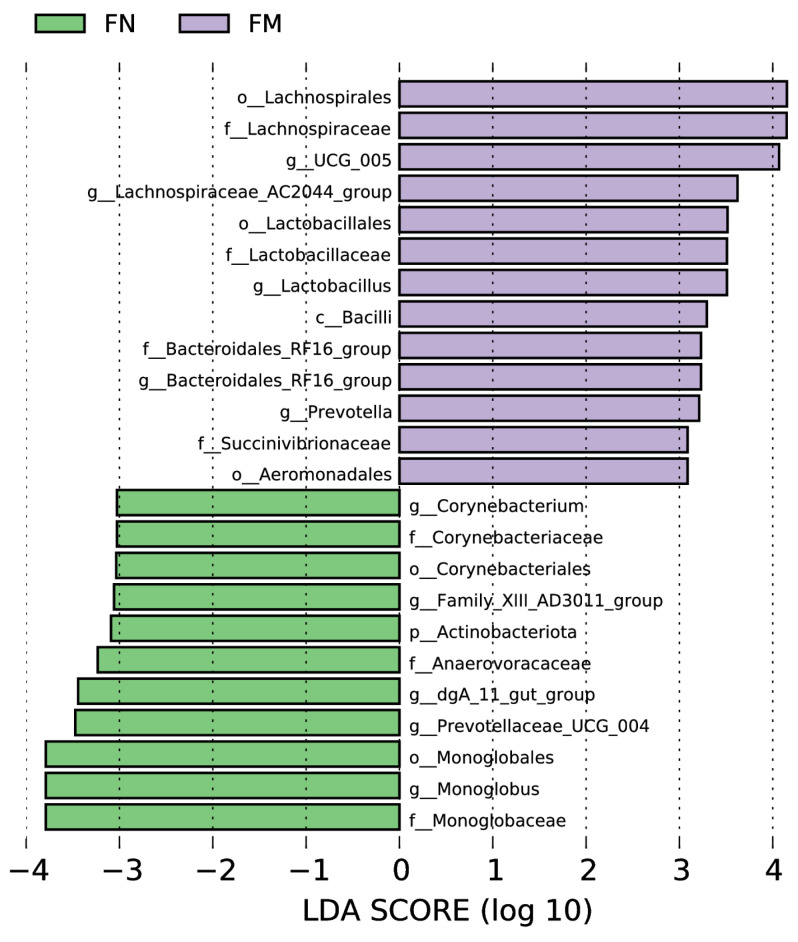
Distinctive bacterial genera in varying treatment cohorts were identified through linear discriminant analysis (LDA) effect size (LEfSe) methodology (LDA score > 4.0).

Biomarker bacteria taxa are shown in Figure 8. We detected bacterial taxa that were predominantly prevalent as distinctive markers among the treatment groups utilizing LEfSe. In total, three significant taxonomic groups (LDA score > 4.0) were recognized, including one genus biomarker. UCG_005 was identified as an indicator species in the Murrah buffalo group.

#### 3.4.3. Correlation of Fecal Microbiota with Fermentation Indices and Milk Fatty Acids Composition

**Figure 9:**
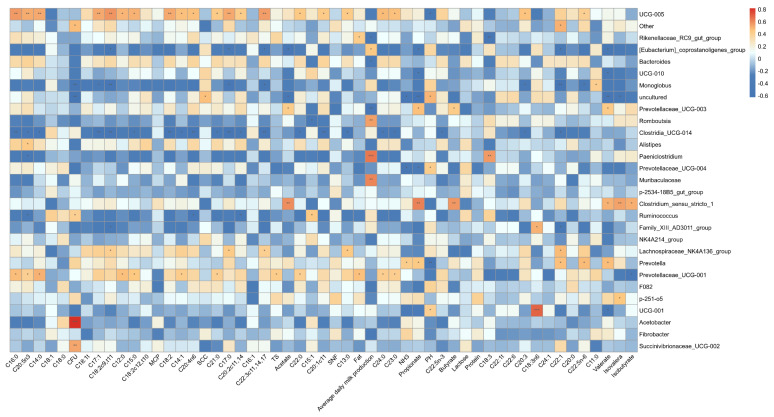
Correlation assessments were performed between the fermentation attributes, milk fatty acid profiles, and the 30 most abundant fecal bacterial genera. In the two-dimensional heatmap visualization, the alteration in the designated color hue and its intensity depicts the kind and magnitude of the correlation, respectively. The asterisk symbol was employed when the r value exceeded 0.1 and the *p* values were below 0.05 (* *p* < 0.05, ** *p* < 0.01, *** *p* < 0.001).

Spearman’s correlation between the relative abundance of fecal bacterial genera and the fermentation characteristics, as well as milk fatty acid contents, is shown in Figure 9. Eighteen milk fat acids levels (C16:0, C20:5n3, C14:0, C17:1, C18:2c9t11, C15:0, C18:2, C14:1, C20:4n6, C21:0, C17:0, C20:2c11 14, C22:3c11 14 17, C22:0, C20:1c11, C13:0, C24:0, and C23:0) showed a positive correlation with UCG-005 but a negative correlation with *Clostrdia*_UCG_004 (*p* < 0.05). *Rikenellaceae*_RC9_gut_group showed a positive correlation with milk fat but a negative correlation with average daily milk production, as well as C18:3 (*p* < 0.05). [*Eubacterium*]_coprostanoligenes_group showed a positive correlation with average daily milk production but a negative correlation with C18:2c9t11, C17:0, C22:3c11 14 17, acetate, propionate, C22:1, and valerate (*p* < 0.05). *Bacteroides* showed a negative correlation with average daily milk production (*p* < 0.05). UCG_010 showed a negative correlation with propionate (*p* < 0.05). *Monoglobus* showed a positive correlation with C12:0 but a negative correlation with C17:1, C18:2c9t11, C17:0, C22:3c11 14 17, C20:3, C22:1, and C22:5n6 (*p* < 0.05). *Prevotellaceae*_UCG_003 and *Clostrdium*_sensu_stricto_1 showed positive correlations with acetate, propionate, butyrate, and valerate (*p* < 0.05). *Romboutsia* showed a positive correlation with average daily milk production but a negative correlation with C15:1 (*p* < 0.05). *Alistipe* and *Paeniclostridium* showed positive correlations with C20:5n3 (*p* < 0.05). *Prevotellaceae*_UCG_004 showed a positive correlation with pH but a negative correlation with NH_3_-N and propionate (*p* < 0.05). *Ruminococcus* showed a positive correlation with C16:1 but negative correlations with C20:5n-3, C20:4n6, and C20:2c11 14 (*p* < 0.05). Family_Xlll_AD3011_group showed negative correlations with C18:2c9t11 and C22:1 (*p* < 0.05). *Lachnospiraceae*_UCG_001 showed positive correlations with C18:2c9t11, C17:0, C22:3c11 14 17, C13:0, and C22:1 (*p* < 0.05). *Prevotella* showed a negative correlation with pH but was positively correlated with propionate, C22:1, C22:5n6, and valerate (*p* < 0.05). *Prevotellaceae*_UCG-001 showed a negative correlation with isobutyrat but positive correlations with C16:0, C20:5n3, C14:0, C12:0, C15:0, C14:1, C21:0, C22:0, C22:0, C23:0, milk fat, and TS (*p* < 0.05). UCG_001 showed a negative correlation with valerate but a positive correlation with pH (*p* < 0.05).

## 4. Discussion

### 4.1. Differences in the Milk Composition of Different Breeds

Breed, feed composition, season of milk production, place of origin, and lactation stage all affect the concentration of fatty acids in fresh milk [40,41,42]. Many studies also have shown that changing the composition of the feed affects the fatty acid composition of milk [42,43]. Matome et al. found that dry matter intake was positively correlated with milk production [44]. Some of fatty acids present in fresh milk are obtained from the rumen fermentation, this is the reason why the objective of this study was to explore the role of gastrointestinal microbiota on milk fatty acids such as C18. Some studies have shown that odd-numbered chain and branched-chain fatty acids are biomarkers of rumen fermentation products, and rumen microorganisms hydrogenate unsaturated fatty acids, generally polyunsaturated fatty acids, usually C18, which is considered as a detoxification process [45]. However, Abdel-Hamid et al. [46] found that there was no significant difference in fat, lactose, and TS content between Murrah and Nili-Ravi buffaloes in mid-lactation conditions during the winter, whereas milk protein and SNF content were significantly greater in Murrah buffaloes than in Nili-Ravi buffaloes. The present study shows that there is no difference in the composition of buffalo milk between the two groups under the same diet. Sun et al. [47] reported that there is no significant difference between Murrah and Nili-Ravi buffaloes for individual fatty acids except C6:0. Abdel-Hamid et al. [46] found that the Murrah buffalo milk fat exhibited greater contents of C18:0, C18:1, C18:2c9t11, C20:1, and α-linolenic acids. In the present study, C18:3, C18:2c9t11, and C22:5n-6 were significantly greater, while C11:0, C18:0, and C18:1 were significantly lower, in milk of Murrah buffaloes, which is partially similar to the findings of Abdel-Hamid et al. [46], and totally different from those of Sun et al. [47]. These differences may be due to the stage of lactation and other reasons. Excessive intake of saturated fatty acids tends to induce inflammation [48] and cause diseases such as high blood lipids and blood pressure [49], while unsaturated fatty acids are beneficial to health, especially conjugated linoleic acid (CLA) in buffalo milk, which can prevent many diseases [14]. Consequently, the fatty acid composition of Murrah buffalo milk is superior to that of Nili-Ravi buffaloes and is more conducive to good health.

### 4.2. Differences in Gastrointestinal Microorganisms

It is important for ruminants to maintain rumen health and internal environmental stability. Ruminal pH, ranging from 5.5 to 7.5, is an important indicator of the health of the rumen environment [50,51], and the rumen pH registered in this experiment was within this range. Volatile fatty acids (acetate, propionate and butyrate) are the main source of energy for ruminants. Acetate is a precursor substance for fatty acid synthesis in ruminants, but acetate also implies a greater production of hydrogen and methane, leading to a lower energy efficiency of fermentation [52]. Propionate can increase blood glucose levels to produce more energy through gluconeogenesis [53]. Research has shown that a lower acetic acid/propionic acid ratio increases the digestibility of dietary fiber. Methane production is highly correlated with the acetate-to-propionic acid ratio over a pH range of 6.5 to 5.3. Furthermore, lower acetic acid/propionic acid ratios result in lower methane production, which is beneficial for energy utilization [51,54]. In this experiment, acetate, propionate and total volatile fatty acids were significantly greater in Murrah buffaloes, but the ratio of acetate to propionate was significantly lower than in Nili-Ravi buffaloes, indicating that fermentation efficiency of Murrah buffaloes is greater.

Lipid metabolism in ruminants generally occurs in the rumen [55] and lipids in the rumen undergo two main processes via mastication and microbiota activity: lipolysis and biohydrogenation into fatty acids [21]. Rumen microbial diversity was not different between breeds, whereas fecal microorganisms were more abundant in Nili-Ravi buffaloes. Ruminal and fecal microorganisms were similar in composition at the phylum level and consisted mainly of *Bacteroidetes* and *Firmicutes*. *Bacteroidetes* were the most abundant rumen microorganisms, followed by *Firmicutes*. On the contrary, in feces, *Firmicutes* were found to account for a greater proportion of the population, followed by *Bacteroidetes.* This result is consistent with many other reports [37,56]. However, there are differences in genera between rumen and fecal microorganisms. Several studies [57,58] found that *Bacteroidetes* can degrade a number of non-fibrous materials and soluble polysaccharides in the rumen of herbivores. *Verrucomicrobiota* have been shown to utilize more complex polysaccharides [58,59], and Proteobacteria are also involved in biofilm formation and the digestion of soluble carbohydrates [60]. Thus, both groups had good carbohydrate digestion, but Nili-Ravi buffaloes could initially digest some polysaccharides from cellulose. *Butyrivibrio* and *Ruminococcaceae* are the main bacteria that digest cellulose, the main component of plant cell walls [61]. *Ruminococcaceae* are the main cellulolytic bacteria producing propionate [62], and *Ruminococcaceae* was also responsible for producing short-chain fatty acids [63]. In the present study, it was found that volatile fatty acids such as propionic acid were greater in the rumen fluid of the Murrah group, which may be due to the higher number of rumen cellulose-degrading bacteria. *Prevotella* are a group of multifunctional bacteria mainly involved in rumen sugar hydrolysis and protein hydrolysis processes [64,65,66]. Therefore, high levels of *Prevotella* in the rumen of both groups are necessary for the degradation of dietary nutrients. Wang et al. [67] showed that *Prevotellaceae*_YAB2003_group digests hemicellulose or xylan more readily. In the present study, we found that *Prevotellaceae*_YAB2003_group was more abundant in Murrah buffaloes, and C18:3 was positively correlated with the abundance of *Prevotellaceae*_YAB2003_group, which may also be one of the reasons for the high content of C18:3 in buffalo milk in Murrah buffaloes. Several of the above bacteria allow carbohydrates to be digested and broken down into volatile fatty acids, which are converted into precursor substances for fat synthesis [68,69,70].

Volatile fatty acids such as acetic acid and propionic acid in the rumen are the precursor substances for the synthesis of long-chain fatty acids in tissues. Therefore, mammary gland tissue can also use them to synthesize long-chain fatty acids and transfer them to milk. Increasing volatile fatty acids such as acetic acid and propionic acid in the rumen will significantly increase the content of C16 and other fatty acids in milk fatty acids [71]. The significant increase in the total volatile fatty acids in the Murrah group compared to the Nili-Ravi buffaloes may be attributed to the greater level of *Acetobacter* in Murrah buffaloes. It has been shown that *Acetobacter* is the main bacterium producing acetate in the rumen [72]. From the Spearman’s analysis in this experiment, we can see that *Acetobacter* is positively correlated with acetate, NH_3_-N, propionate, butyrate, C22:5n-6, and valerate, and negatively correlated with pH. Therefore, we speculated that the higher content of volatile fatty acids such as propionic acid and C22:5n-6 in the Murrah buffaloes might also be related to the higher content of *Acetobacter* in the Murrah buffaloes. In contrast, in our study, we found that acetate, NH_3_-N, propionate, butyrate, and isovalerate were negatively correlated with *Prevotellaceae*_UCG_003 (*p* < 0.05), and *Prevotellaceae*_UCG_003 was significantly greater in Nili-Ravi buffaloes than in Murrah buffaloes, which may also be one of the reasons for the lower content of indicators such as acetic acid in Nili-Ravi buffaloes. The higher fatty acid concentration of buffalo milk in Murrah buffaloes may be attributed to the fact that buffaloes of this breed are able to utilize these volatile fatty acids in the rumen more efficiently by converting them into long-chain fatty acids through the biosynthetic pathway. The lipids then produce a series of intermediates such as C18:1, C18:1t11, and conjugated linoleic acid and its isomers due to incomplete hydrogenation by rumen microbes [73]. In this experiment, the quantity of unsaturated fatty acids such as C18:2c9t11, C18:2c12t10, and C18:1 was significantly greater in the rumen of the Murrah buffaloes, whereas saturated fatty acid content was significantly lower. This may be due to the fact that other less abundant bacteria have biohydrogenated the lipids. At the same time, a similar trend was observed for milk fatty acids composition, and it can be confirmed that the composition and content of rumen fatty acids also have an important effect on milk fatty acid composition.

The fecal microbiota of the Murrah buffalo has a relatively abundant content of UCG_005, which belongs to the family *Ruminococcaceae*. UCG_005 was positively correlated with linoleic acid as well as conjugated linoleic acid, suggesting that UCG_005 from *Ruminococcaceae* may be associated with lipid metabolism. This has been consistently described in both chicken and bovine studies [74,75]. The abundance of UCG_005 in the Murrah group was significantly higher than that in the Nili-Ravi group, and the genera biomarker in the Murrah group was also UCG_005, which could also explain the higher levels of linoleic acid as well as conjugated linoleic acid in the Murrah group.

## 5. Conclusions

In general, the levels of unsaturated fatty acids in Murrah buffalo milk were greater than those in Nili-Ravi buffalo milk, which was consistent with the concentration of unsaturated fatty acids in the rumen fluid. Moreover, the concentration of volatile fatty acids in the rumen of Murrah buffalo was also relatively high. In addition, the abundance of bacteria in the rumen and feces of Murrah buffalo, which positively correlated with unsaturated fatty acids, was higher. Therefore, we can regulate the fatty acid composition in buffalo milk by adjusting the composition of gastrointestinal microbiota.

## Figures and Tables

**Table 1:** Constituents and nutrient concentrations in basal diet of water buffaloes.

Ingredients	Dry Matter, %
Corn	8.95
Soybean meal	2.88
Beer residue	26.56
White wine lees	7.50
Pineapple peel	13.96
Elephant grass silage	18.75
Sodium cyclamate	5.00
Peanut vine	9.00
Stevia	2.50
Wheat bran	3.46
NaCl	0.23
NaHCO_3_	0.40
CaHPO_4_	0.25
CaCO_3_	0.30
Additive ^1^	0.07
Premix ^2^	0.19
Chemical components, %	
Dry matter (DM)	35.86
Crude protein (CP)	18.21
Ether extract (EE)	3.97
Neutral detergent fiber (NDF)	38.51
Acid detergent fiber (ADF)	22.05
Crude ash (ASH)	8.24

^1^. Mildew removers and preservatives. ^2^. For each premix containing: VE 3 000 IU, VD 150 000 IU, VA 500 000 IU, Cu 1.3 g, Fe 4.0 g, Mn 3.0 g, I 80 mg, Zn 6.0 g, Co 80 mg, Se 50 mg, calculated based on theoretical values.

**Table 2:** Effects of buffaloes’ breed on milk yield and milk composition.

Breed	Average Dry Matter Intake (kg/d)	Milk Yield (kg/d)	Milk Protein (%)	Milk Fat (%)	Lactose (%)	Total Solid (TS, %)	Solids Non-Fat (SNF, %)
Nili-Ravi buffalo	10.07 ± 0.37	8.60 ± 1.29	4.45 ± 0.46	8.26 ± 1.23	5.22 ± 0.15	19.12 ± 1.73	10.00 ± 0.51
Murrah buffalo	10.26 ± 0.29	7.88 ± 1.40	4.69 ± 0.33	9.13 ± 1.09	5.12 ± 0.28	20.27 ± 1.52	10.23 ± 0.50
*p*-value	0.607	0.272	0.165	0.091	0.336	0.112	0.314

**Table 3:** Effect of buffalo breed on milk fatty acids concentration (mg/100 g milk fat).

Fatty Acid	Nili-Ravi Buffalo	Murrah Buffalo	*p*-Value
C11:0	1.27 ± 0.22	0.99 ± 0.26	0.032
C12:0	217.97 ± 126.77	334.11 ± 152.71	0.065
C13:0	8.77 ± 5.37	12.13 ± 6.72	0.209
C14:1	86.79 ± 53.89	121.57 ± 68.52	0.199
C14:0	1679.54 ± 785.02	1975.84 ± 841.19	0.402
C15:1	10.62 ± 5.95	13.61 ± 9.32	0.427
C15:0	206.13 ± 115.63	229.97 ± 117.18	0.636
C16:1	25.16 ± 22.86	23.18 ± 20.62	0.858
C16:0	3577.65 ± 1106.44	3589.67 ± 1086.30	0.980
C17:1	285.85 ± 152.71	376.87 ± 240.38	0.301
C17:0	33.75 ± 18.06	49.56 ± 38.85	0.234
C18:3n6	2.36 ± 1.08	2.37 ± 0.81	0.981
C18:2	113.92 ± 65.01	136.07 ± 81.09	0.487
C18:3	2.50 ± 0.90	4.07 ± 1.82	0.044
C18:2c9t11	266.25 ± 110.75	438.66 ± 212.54	0.037
C18:1	1572.06 ± 620.68	955.94 ± 352.19	0.038
C18:2c12t10	231.28 ± 150.47	219.87 ± 175.58	0.872
C18:1t	433.39 ± 278.39	585.40 ± 333.98	0.259
C18:0	1171.43 ± 196.01	899.13 ± 158.18	0.027
C20:4n6	72.02 ± 33.91	81.09 ± 43.99	0.617
C20:5n3	1975.78 ± 765.05	1934.19 ± 675.14	0.894
C22:3c11,14,17	18.26 ± 6.34	25.75 ± 10.12	0.124
C20:3	2.34 ± 1.55	3.90 ± 2.76	0.117
C20:1c11	13.57 ± 8.74	14.14 ± 7.64	0.873
C20:2c11,14	29.85 ± 25.49	30.79 ± 19.7	0.924
C20:0	2.45 ± 1.66	2.35 ± 1.23	0.871
C21:0	64.65 ± 42.81	65.90 ± 37.56	0.943
C22:6	4.73 ± 8.76	1.96 ± 1.40	0.313
C22:5n-3	5.82 ± 4.12	6.00 ± 3.54	0.911
C22:5n-6	1.16 ± 0.56	2.05 ± 1.27	0.045
C22:1	1.80 ± 0.95	3.39 ± 3.34	0.144
C22:1t	5.24 ± 11.64	1.85 ± 0.89	0.347
C22:0	14.86 ± 11.24	16.22 ± 9.96	0.768
C23:0	7.86 ± 5.83	9.44 ± 6.00	0.536
C24:1	1.20 ± 0.47	1.27 ± 0.44	0.733
C24:0	8.00 ± 6.07	10.05 ± 6.56	0.457
Saturated fatty acid (SFA)	6374.57 ± 2309.23	6770.82 ± 2541.65	0.706
Unsaturated fatty acid (UFA)	5731.45 ± 2208.76	5329.84 ± 1373.62	0.614
Monounsaturated fatty acid (MUFA)	2298.65 ± 842.15	1688.92 ± 768.98	0.090
Polyunsaturated fatty acid (PUFA)	3432.80 ± 1659.16	3640.92 ± 960.35	0.722
SFA/UFA	1.20 ± 0.39	1.27 ± 0.35	0.623
Total fatty acid	12,106.02 ± 3985.09	12,100.66 ± 3635.53	0.997

**Table 4:** Effect of buffalo breed on rumen fermentation parameters.

Items	Nili-Ravi Buffalo	Murrah Buffalo	*p*-Value
pH	6.79 ± 0.18	6.41 ± 0.3	0.001
Ammonia nitrogen (NH_3_-N, mg/100 mL)	6.02 ± 1.85	9.34 ± 3.67	0.013
Microbial protein (MCP, mg/mL)	0.12 ± 0.03	0.13 ± 0.02	0.637
Acetate (mmol/L)	13.96 ± 1.89	16.38 ± 2.74	0.027
Propionate (mmol/L)	6.63 ± 1.07	8.58 ± 2.09	0.011
Isobutyrate (mmol/L)	0.15 ± 0.05	0.16 ± 0.06	0.523
Butyrate (mmol/L)	4.91 ± 0.87	5.61 ± 1.34	0.158
Isovalera (mmol/L)	0.29 ± 0.08	0.38 ± 0.12	0.049
Valerate (mmol/L)	0.60 ± 0.13	0.73 ± 0.15	0.033
Total volatile fatty acid (TVFA, mmol/L)	26.55 ± 3.92	31.31 ± 6.60	0.051
Acetate/Propionate	2.11 ± 0.12	1.88 ± 0.21	0.003

**Table 5:** Influence of buffalo breed on the ruminal fatty acid concentrations (μg/mL).

Ruminal Fatty Acid	Nili-Ravi	Murrah	*p*-Value
C11:0	0.12 ± 0.09	0.14 ± 0.09	0.646
C12:0	2.05 ± 0.61	1.39 ± 0.51	0.024
C13:0	0.22 ± 0.09	0.15 ± 0.09	0.060
C14:0	2.81 ± 0.71	3.12 ± 0.96	0.425
C15:1	0.58 ± 0.46	0.25 ± 0.14	0.164
C15:0	2.16 ± 0.69	3.52 ± 0.92	0.005
C16:1	1.03 ± 0.17	0.91 ± 0.28	0.275
C16:0	100.94 ± 3.42	100.24 ± 17.50	0.927
C17:1	0.43 ± 0.06	0.33 ± 0.06	0.001
C17: 0	0.52 ± 0.21	0.83 ± 0.19	0.006
C18:3n6	0.53 ± 0.21	0.65 ± 0.32	0.213
C18:2	0.46 ± 0.20	1.33 ± 0.39	0.001
C18:3	2.12 ± 0.70	1.01 ± 0.55	0.008
C18:2c9,t11	6.41 ± 0.98	9.00 ± 2.77	0.034
C18:1	7.02 ± 2.35	7.65 ± 1.49	0.521
C18:2t10,c12	3.07 ± 0.74	4.88 ± 1.39	0.006
C18:1t	0.19 ± 0.13	17.60 ± 2.48	0.001
C18:0	6.91 ± 3.71	1.85 ± 0.72	0.028
C20:4n6	0.32 ± 0.17	0.65 ± 0.35	0.019
C20:5n-3	114.09 ± 15.25	137.58 ± 33.31	0.099
C22:3	0.31 ± 0.07	0.14 ± 0.05	0.001
C20:3	0.29 ± 0.12	0.17 ± 0.08	0.028
C20:1	0.21 ± 0.09	0.15 ± 0.09	0.152
C20:2	0.32 ± 0.13	0.64 ± 0.31	0.013
C20:0	0.25 ± 0.08	0.16 ± 0.08	0.017
C21:0	1.35 ± 0.30	1.93 ± 0.73	0.037
C22:6	0.17 ± 0.09	0.13 ± 0.08	0.362
C22:5n-3	0.20 ± 0.11	0.23 ± 0.09	0.422
C22:5n-6	0.19 ± 0.08	0.17 ± 0.10	0.607
C22:1	0.20 ± 0.15	0.20 ± 0.08	0.875
C22:1t	0.22 ± 0.12	0.17 ± 0.11	0.316
C22:0	0.19 ± 0.07	0.28 ± 0.14	0.074
C23:0	0.15 ± 0.12	0.13 ± 0.07	0.651
C24:1	0.21 ± 0.12	0.16 ± 0.08	0.308
C24:0	0.22 ± 0.11	0.36 ± 0.18	0.037
Omega-3 ^1^	120.89 ± 39.57	163.46 ± 51.90	0.041
Omega-6 ^2^	1.14 ± 0.48	1.88 ± 0.95	0.030
Omega-9 ^3^	9.82 ± 6.70	19.53 ± 8.26	0.010
Saturated fatty acid (SFA)	98.21 ± 28.80	120.83 ± 36.21	0.119
Unsaturated fatty acid (UFA)	146.27 ± 47.28	200.74 ± 63.65	0.033
Monounsaturated fatty acid (MUFA)	14.63 ± 8.72	25.88 ± 12.08	0.020
Polyunsaturated fatty acid (PUFA)	131.63 ± 41.27	174.86 ± 54.44	0.051
SFA/UFA	0.68 ± 0.06	0.61 ± 0.05	0.001
Total fatty acid	244.48 ± 75.28	321.57 ± 99.03	0.051

^1^ Omega-3 includes C18:2c9t11, C18:2t10c12, C20:5n3, and C22:5n3; ^2^ Omega-6 includes C18:3n6, C20:4n6, and C22:5n6; ^3^ Omega-9 includes C18:1, C18:1t, C20:3, C20:1, C22:1, and C24:1.

## Data Availability

The sequence data generated in this experiment (16SrRNA gene sequences) were deposited in SRA database of NCBI under Bioproject No. PRJNA1010523.
